# Regionalization of gene expression and cell types in the silk glands of *Plodia* pantry moths

**DOI:** 10.1016/j.isci.2025.113865

**Published:** 2025-10-25

**Authors:** Jasmine D. Alqassar, Mathilde Biot, Lauren E. Eccles, Whitney L. Stoppel, Arnaud Martin

**Affiliations:** 1 Department of Biological Sciences, The George Washington University, Washington, DC 20052, USA; 2 Department of Meiosis, Max Planck Institute for Multidisciplinary Sciences, 37007 Göttingen, Germany; 3 Department of Chemical Engineering, University of Florida, Gainesville, FL 32603, USA

**Keywords:** Biological sciences, Entomology, Molecular biology

## Abstract

Lepidopteran silk glands purportedly include a posterior silk gland (PSG) that secretes core fibers and a middle silk gland (MSG) that secretes adhesive sericins. While well-studied in the silkworm, little is known about other silk glands. Here, we used quantitative and spatial assays to profile gene expression in the silk glands of the pantry moth *Plodia interpunctella*. RNA-seq revealed the transcriptomic divergence between MSG and PSG. High expression of fibroin genes *FibL* and *FibH* was specific to the PSG, whereas the MSG was dominated by the expression of major sericins—bioadhesive proteins that coat the silk fiber. Hybridization chain reaction (HCR) mRNA profiling resolved sharp cellular boundaries within the silk gland: PSG cells expressed fibroins, while MSG cells comprised two compartments, each expressing different sericins. Our findings underscore the organization of lepidopteran silk glands into specialized secretory subdivisions and establish *Plodia* as a promising comparative model for studying silk diversity.

## Highlights

• The pantry moth is introduced as a model for silk gland biology
• RNAseq identifies fibroin and sericin genes as major silk proteins
• Hybridization chain reactions map RNA expression with cellular precision
• Findings shed light on conserved silk gland compartmentalization in Lepidoptera

## Graphical abstract

**Figure:**
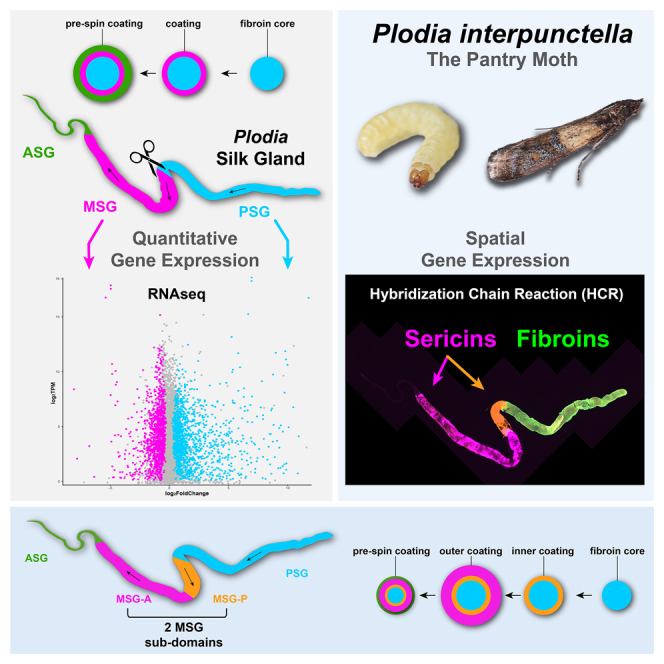

## Introduction

There are an estimated 180,000 species of moths and butterflies (Lepidoptera), and most produce silk throughout their larval life, with common functions including locomotion, protection, habitat construction, and pupation. Silk is a complex biomaterial formed through protein self-assembly in a pair of labial glands (the silk glands), where silk proteins are secreted and then extruded through the spinneret near the mouth.^1^^,^^2^ In Lepidoptera, silk fibers are characterized by a fibroin core with an outer protein coating.^3^^,^^4^^,^^5^ The inner fibroin core provides the primary structural strength and toughness of the silk fiber,^6^^,^^7^^,^^8^^,^^9^ while the outer coating modulates properties such as hydrophilicity and adhesion.^5^^,^^10^^,^^11^^,^^12^^,^^13^ Each lepidopteran organism that produces silk fibers regulates the composition of these layers through the expression of secreted proteins in its silk glands.^4^

Investigations in *Bombyx mori* have outlined the general structure of the silk gland as divided into three distinct regions, each with specialized functions related to silk production.^5^^,^^14^^,^^15^ The posterior silk gland (PSG) is primarily responsible for the production and accretion of proteins that form the core of the silk fiber: Fibroin Heavy chain (FibH), Fibroin Light chain (FibL), and Fibrohexamerin (Fhx, also known as P25^16^). FibH contains repetitive motifs that create crystalline domains and underlie the fiber’s mechanical strength.^1^^,^^9^^,^^17^^,^^18^^,^^19^^,^^20^^,^^21^ The C-terminal region of FibH forms covalent bonds with FibL, which acts as an essential linker to form silk crystalline units.^22^^,^^23^ Fhx is dispensable for silk production but is thought to assist with the trafficking of the fibroin proteins before they accumulate within the lumen of the gland.^24^^,^^25^ The middle silk gland (MSG) is primarily responsible for the synthesis of sericin proteins, a family of hydrophilic adhesive proteins that form a coating layer around the fibroin core.^11^ In the silkworm *Bombyx mori*, gland morphology and sericin expression profiles of the silkworm MSG suggest it is itself subdivided into three subregions called the anterior, middle, and posterior MSGs, abbreviated MSG-A, MSG-M, and MSG-P.^26^^,^^27^^,^^28^^,^^29^^,^^30^ Last, the anterior silk gland (ASG) consists of a thin duct involved in silk fiber alignment and stretching before extrusion through the spinneret.^26^^,^^27^^,^^31^ The ASG is also connected to a small pair of exocrine structures called Filippi’s glands,^32^ and this accessory gland likely contributes to silk maturation and cocoon compaction in *Bombyx*.^33^

While the silk of the domesticated silkworm *Bombyx mori* has been extensively characterized, it represents only a single lineage within the vast diversity of Lepidoptera, and expanding this research to more species holds great promise to uncover the proteins that underlie the mechanical properties,^34^ adhesivity,^25^ and antimicrobial activity of silks specialized for different ecological uses.^35^^,^^36^^,^^37^ This comparative endeavor has been spurred by the generation of silk gland transcriptomes in an increasing number of lepidopteran lineages.^38^^,^^39^^,^^40^^,^^41^^,^^42^^,^^43^^,^^44^^,^^45^^,^^46^^,^^47^^,^^48^^,^^49^ However, few of these species benefit from state-of-the-art gene annotation resources, and because spatial assays that directly visualize gene products in dissected glands have been limited to *Bombyx*,^12^^,^^17^^,^^28^^,^^29^^,^^30^^,^^31^^,^^32^^,^^33^ these studies often offer a limited resolution on how gene expression varies in the silk gland.

The pantry moth, *Plodia interpunctella*, is a promising alternative model system for the study of silk biology, as recent work has highlighted its potential in silk fiber production for biotechnology applications,^50^^,^^51^^,^^52^ as well as a laboratory powerhouse for genetic manipulation.^53^^,^^54^^,^^55^^,^^56^ To expand the understanding of the structure and organization of the *Plodia* silk gland, we carried out an RNAseq differential gene expression analysis of the MSG and PSG. We selected this species as a model system for silk biology due to the rearability of this insect, its potential for functional genomics,^54^^,^^55^^,^^56^ and the availability of an annotated genome. As a pyralid moth, *Plodia* is also phylogenetically close to *Ephestia kuehniella* and *Galleria mellonella*, where comparative studies of gland histology and gene expression suggest that the MSG subdivides into two domains of sericin expression.^10^^,^^32^ To test this in *Plodia*, we leveraged the spatial specificity of hybridization chain reaction (HCR), a technique that amplifies the fluorescent signal of small probes targeted at an mRNA of interest, and refined the expression patterns of silk factor genes in whole-mount glands with a subcellular resolution. We discuss how combining RNAseq and HCR primes future explorations of silk diversity in Lepidoptera and beyond.

## Results

### Morphology and cellular organization of the *Plodia* silk glands

**Figure 1:**
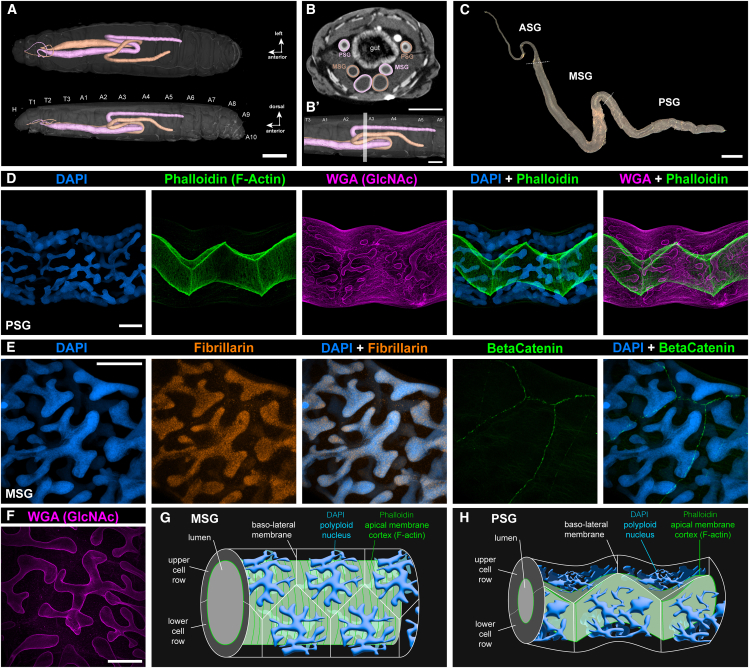
Cellular morphology of *Plodia* silk glands (A) Micro-CT scan of a fifth instar larva with each silk gland rendered in 3D. False coloring marks the identity of each individual glands. Glands switch sides twice in this individual (in T2 and A3), but this crossing does not always occur. Top: ventral view; bottom: lateral view. Animated version in Video S1. (B-B′) Cross-sectional view of a scanned larva in the A3 segment (section plane as shown in B′), where the PSG and two sections of the MSG are visible. Gland outer and inner diameters both increase as the gland progresses toward the ASG. Animated version in Video S2. (C) Dark-field image of a dissected silk gland from a *Plodia* fifth instar wandering larva. Dotted lines indicate the ASG/MSG (top) and MSG/PSG (bottom) boundaries. (D) DAPI, Phalloidin (F-Actin), and WGA (GlcNAc) staining of a wandering fifth instar PSG. Comparisons with ASG and MSG sections are featured in Figure S1 and Video S3. (E) DAPI and monoclonal antibody staining for Fibrillarin (marking nucleoli), and Beta-Catenin (accumulating at apical cell membrane junctions) in a fifth instar *Plodia* MSG. (F) WGA staining of the nuclear envelope in polyploid nuclei of the MSG. (G and H) Schematic representation of cell organization and geometry in the MSG (G), and PSG (H). Scale bars: A = 1 mm; B-B′, C = 500 μm; D-F = 50 μm.

We used dissections and high-resolution micro-computed tomography (micro-CT) of fifth instar larvae to reveal the positioning and overall morphology of the *Plodia* silk glands (Figures 1A–1C). Each gland forms an epithelial tube connected to the mouth spinneret via a thin ASG, followed by thick MSG section resting ventrally to the gut and starting in the T2 mesothoracic segment. The two glands curve backwards when reaching the A4 abdominal segments and then curve back toward the posterior end of the A2 segment. This second curve approximately marks the beginning of the two PSGs, which run in a position lateral to the gut from the A3 to A5 segments.

Video S1. 3D rendering of silk glands in a fifth instar moth larva visualized by micro-CT, related to Figure 1

Video S2. XY-plane micro-CT slices through a fifth instar moth larva Animated sequence of consecutive 2D micro-CT sections scrolling through the larva from anterior to posterior. Slices highlight internal anatomical structures, including the changes in diameter, looping, and crossing of the silk glands along the larva’s body axis, related to Figure 1

Next we visualized the cellular morphology of the PSG and MSG using confocal microscopy (Figures 1D and S1), using DAPI stainings of nuclear DNA, phalloidin stainings of F-actin, and WGA stainings of N-acetylglucosamine (GlcNAc, labeling various membranal features). Phalloidin marked the apical surface of all cells along the silk gland, thus effectively contouring the luminal space left by cells. Packing of the single-cell layer results in 120° angles at the junctions between the three cells, characteristic of epithelial tubes,^57^ in both the PSG and MSG. Apical cell surfaces show an accordion shape in the PSG, likely due to the small size of the lumen and the relatively small diameter of the gland (Figures 1D and S1C, and Video S3). In the MSG, as the lumen has increased in diameter (Figure S1B), the cell apical surfaces that line it up are stretched around its periphery (Figure S1B and Video S3). Apical F-actin formed transverse striations forming ring-like structures around the lumen, perpendicular to the gland longitudinal axis. This overall geometry is consistent with the previously identified cellular morphology of the ASG, MSG, and PSG in silkworms.^58^

Video S3. 3D projection of DAPI, Phalloidin, and WGA stainings, from confocal microscopy stacks of the ASG, MSG, and PSG, related to Figure 1

DAPI stainings show that MSG and PSG nuclei are extremely large and elongated (Figures 1D–1H), with a branching architecture also visible in WGA stainings of the nuclear envelope.^59^ Such large nuclei are typical of silk glands across Lepidoptera and are caused by endoreplication, a mode of cell cycling characterized by genome replication leading to an increase in cell size without cell or nuclear division.^60^^,^^61^^,^^62^^,^^63^ The branching architecture increases the surface-to-volume ratio of nuclei and was proposed to optimize the efficiency of gene expression and transcript processing in these large cells.^63^ Stainings of silk gland nucleoli using an anti-fibrillarin antibody show a constellation of signal puncta (Figure 1E), contrasting with the 1–4 nucleoli expected in diploid cells. While we could not quantify these signals to measure ploidy levels reliably, a previous study of the equivalent cells in *Ephestia* last-instar larvae measured that silk gland cells accumulated over 8,000 genome copies.^63^ Cells and nuclei are prone to bursting due to their enormous size, and we were unable to isolate intact silk gland cells or live nuclei using cell-dissociation methods optimized for less polyploid (4*n*-32*n*) cells from developing pupal wings,^64^^,^^65^ which precluded us from attempting single-cell transcriptomics in this project.

### Hybridization chain reaction reveals a sharp boundary between the MiddleSilk Gland and Posterior Silk Gland

**Figure 2:**
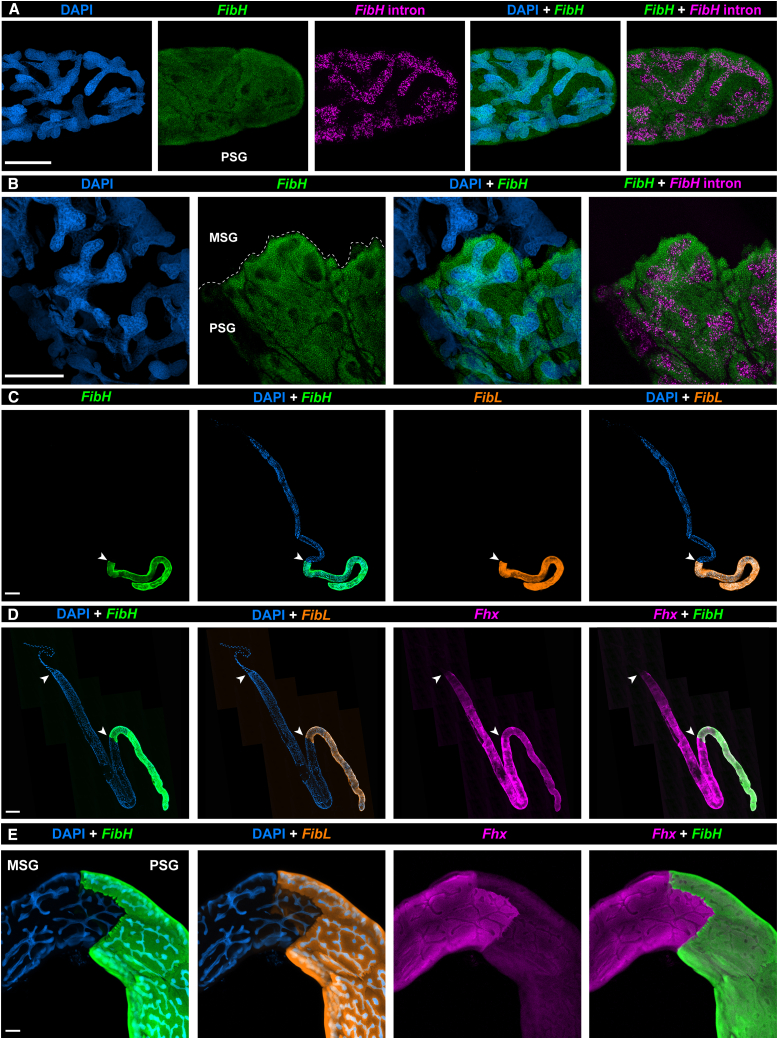
HCR detection of fibroin transcripts reveals tissue boundaries in the silk gland (A and B) HCR stainings using exonic and intronic *FibH* probes in the posterior PSG (A) and at the MSG/PSG interface (B). (C) Whole-mount fifth instar silk gland HCR stainings for *FibH* and *FibL*. (C–E) HCR stainings of wandering fifth instar silk glands for *FibH, FibL* and *Fhx* mRNA. Arrowheads: MSG/PSG (C-D: bottom) and ASG/MSG boundaries (D: top). Scale bars: A-B, E = 50 μm; C, D = 500 μm.

We sought to test the suitability of HCR RNA *in situ* hybridization^66^^,^^67^ to detect gene expression at subcellular resolution within the *Plodia* silk gland, initially targeting the intronic and exonic sequences of fibroin heavy chain (*FibH*) (Table S1). Both probe sets showed a strong signal in PSG cells: the intronic probe signal was restricted to the nucleus, consistent with the detection of nascent transcripts, while exonic probes showed a diffuse signal of the mRNA in the nucleus and cytoplasm (Figure 2A). *FibH* intronic and exonic stainings, as well as fibroin light chain (*FibL* mRNA staining, were exclusive to the PSG and revealed a strong spatial boundary with the adjacent MSG (Figures 2B and 2C). *FibH* and *FibL* mRNA expression colocalizes in the PSG and has prominent boundaries of expression. Unexpectedly, staining for *P25/Fibrohexamerin* (*Fhx*) showed prominent expression in the MSG, a weaker signal in the PSG, and a lack of expression in the ASG, thereby delineating a sharp ASG/MSG boundary between non-exocrine and exocrine sections of the silk gland (Figures 2D and 2E). The expression of *Fhx* throughout the MSG-PSG is consistent with a previous study in *Ephestia* but contrasts with *Bombyx*, where *Fhx* is restricted to the PSG.^10^^,^^67^ Fhx is dispensable for silk fibroin assembly but participates in its secretion in *Bombyx.*^24^ Given its expression in both the MSG and PSG in the closely related *Plodia* and *Ephestia* moths, as well as in bagworms,^41^ Fhx might fulfill a generic, secretory function that extends to the adhesive coat layers produced by the MSG in these species. Overall, these results posit HCR as a powerful spatial assay for profiling gene expression in whole-mount silk glands and highlight sharp functional boundaries in the silk gland.

### RNAseq of the Posterior Silk Gland and Middle Silk Gland uncovers the major silk factors of *Plodia* silk

**Figure 3:**
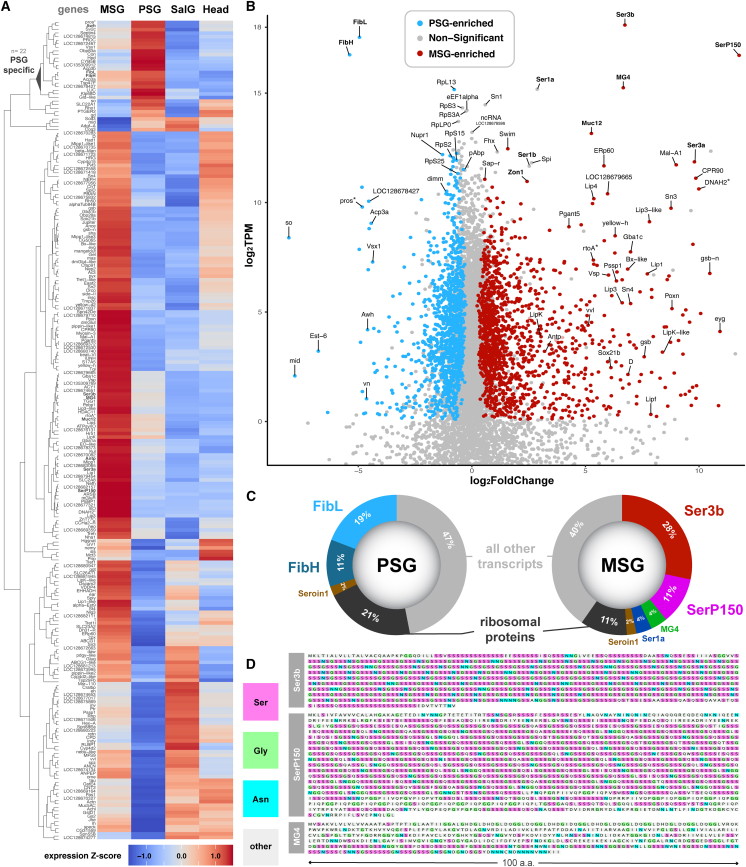
RNAseq profiling of transcriptome divergence in the PSG and MSG specialized glands (A) Heatmap of the top 229 DEGs between MSG and PSG (adjusted *p* < 0.01, |log_2_FoldChange| > 3, DESeq2 normalized counts >300). Average gene expression profiles across tissues are shown with a *Z* score transformation and sorted by hierarchical clustering (SalG: salivary glands; Heads: larval heads). A version of this heatmap with individual samples’ expression profiles is shown in Figure S2. (B) Scatterplot highlights expression level difference of individual genes between the PSG and MSG (x axis, log2FC), and their relative transcript abundance within their tissue of enrichment (y axis, log_2_TPM). Coloring highlights genes with DESeq2 adjusted *p* < 0.05 and log_2_TPM >0.01. Non-significant, lowly expressed genes (log_2_TPM < −2) are not shown. Asterisks: the genes *prospero* (*pros*), *rtoA*, and *DNAH2* are respectively adjacent to DEGs *FibH, MG4*, and *SerP150*, which may drive their enrichment in the corresponding tissues. (C) Transcript representation within the PSG and MSG tissues (TPM as %). Ribosomal protein genes were pooled. (D) Amino acid composition of three major secreted proteins detected in the MSG, each showing extensive serine-rich stretches and repeats characteristic of sericin proteins. Accession numbers: GenBank: XP_053622673 (Pi_Ser3b), GenBank: XP_053622719 (Pi_MG4), GenBank: XP_053613126 (Pi_SerP150).

Next, we generated deep transcriptomes of the MSG and PSG of fifth instar larvae, as well as salivary glands and whole larval heads from the same individuals (four biological replicates per tissue, 16 libraries total). Dissected silk glands were manually split at the approximate MSG/PSG boundary using fine dissecting scissors before RNAseq, resulting in A/MSG samples (MSG, including portions of the ASG, but not the Filippi’s glands) and PSG samples. A/MSG transcriptomes include a contribution from ASG cells, but given the small size of this tissue and cells (Figures 1 and S1), we henceforth refer to these samples as pertaining to the MSG for simplicity, as the ASG should have a relatively minor impact on overall expression. In order to increase the statistical power of differential expression analyses, salivary gland samples were included as an exocrine tissue not involved in silk production, and larval heads as a control encompassing a heterogeneous mixture of cell types. Principal component analysis found that variation among tissue replicates was minimal and tissue types clustered together as expected; additionally, the MSG and PSG clusters were closer to each other than the salivary gland and head tissue clusters (Figure S2A). DESeq2 analysis detected a total of 2,914 differentially expressed genes (DEGs) between the MSG and PSG (Table S2; adjusted *p* < 0.05). GO enrichment analysis showed enriched genes have a bias toward transcriptional and translational processes (Figure S2C). Out of the 229 top DEGs—defined here as those with the highest expression differences (log_2_FC > 3)—22 were specific to the PSG (Figures 3A and S2B; Tables S2, S3, and S4), including *FibH*, *FibL,* and *Arrowhead* (*Awh*), a transcription factor known to regulate fibroin genes in silkworms.^68^^,^^69^ Out of 229 top DEGs, 195 were enriched in the MSG relative to the PSG (Figure 3A; Tables S2, S3, and S4). Among them, genes that have been associated with the MSG of pyralid moths such as *Ser3a*, *Mucin12*, *SerP150*, *MG4, and Zon1*,^10^^,^^12^^,^^70^ as well as the homeobox gene *Antp,* known to determine the MSG identity in silkworms,^29^^,^^30^^,^^45^ were restricted to the MSG.

We hypothesized that secreted silk factors are expressed at higher levels than non-secreted products. In addition to fold-change expression differences between the MSG and PSG, we used mean TPM (transcripts per million reads) values as a measure of transcript abundance within tissues^71^ (Figure 3A; Table S5). A scatterplot summarizes this combination of relative expression within tissues and fold-change expression differences between tissues (Figure 3B; Table S6). Both transcriptomes were strongly biased toward a small set of highly expressed secreted factors (Figure 3C): *FibH* and *FibL* are by far the two highest expressed genes in the PSG transcriptome, amounting to 30% of all transcript molecules sequenced in this tissue, and four sericin genes named *Ser3b*, *SerP150, MG4* and *Ser1a* (Figures 3D and S2–S6; Table S7) represent 49% of the transcript molecules sequenced in the MSG. *Ser3b* alone accounts for 28% of all transcript molecules detected in the MSG, making this paralog of *Ser3a* (Figure S6) a major component of the *Plodia* silk coating layer. Three other sericin genes (*Ser1b*, *Ser3a,* and *Mucin12*) were also detected in the MSG at lower levels (0.3–0.9%).

Both PSG and MSG tissues showed high expression of ribosomal proteins, which together constitute 21% and 11% of the PSG and MSG transcriptomes, as well as of the protein elongation factors *eEF1alpha1*, *eEF1beta*, *eEF1gamma*, *eEF2*, *eEF5,* and the *pAbp* translation termination factor, each with a log_2_TPM >10 (Figure 3C; Tables S5 and S6). Of note, both the PSG and MSG also expressed the seroin gene *Seroin1* (*Sn1*, log_2_TPM >14.4) as well as a silk gland-specific non-coding RNA of unknown function (*LOC128679596*, log_2_TPM >12.7). With the exception of their main fibroin and secreted factors, the most highly expressed transcripts were thus shared between the MSG and PSG.

### Candidate determinants of Anterior Silk Gland/Middle Silk Gland/Posterior Silk Gland specialization at transcriptional and post-translational levels

Candidate genes for the regulation of transcription showed marked divergence between the PSG and MSG, some of which likely underlie the partitioning of the silk gland into at least two distinct exocrine tissues. Indeed, several transcription factors (TFs) were differentially expressed between the two regions and may be important regulators of their identity (Figure 3B). In the PSG, this included genes encoding Awh and Dimmed, both known to activate fibroin gene expression in silkworm^68^^,^^69^^,^^72^^,^^73^; Vsx1, a homeobox TF that binds the *FibH* promoter directly in silkworms^48^; as well as Midline and Sine oculis, for which a function in the PSG remains to be studied. Among MSG-enriched TF factors, Antp and Gsb are known to impact MSG identity and morphology in silkworms.^29^^,^^30^^,^^45^^,^^74^ Previous research in silkworms has shown that Gsb is a key enforcer of ASG and MSG identity, as it is responsible for both transcriptional repression of fibroin gene expression and symmetrical cell growth, necessary qualities for sufficient silk production and spinning.^58^^,^^74^^,^^75^ We also detected additional genes encoding the TFs Bx-like, Vvl, Eyegone (Eyg), Poxn, Gsb-N, Dichaete (D), and Sox21b that may contribute to the specialization of the MSG transcriptome.

Second, the specialization of each region may involve enzymes that modify the secreted content. Pyralid sericins are water soluble, glue-like proteins that likely undergo post-translational modifications.^12^^,^^76^ The gene *Pgant5* showed a strong enrichment in the MSG (Figures 3A and 3B). In *Drosophila*, Pgant5 performs the first enzymatic step leading to the addition of N-acetylgalactosamine (GalNAc) sugar to Serine/Threonine (Ser/Thr) residues in the gut and salivary glands, a process known as mucin-type O-glycosylation.^77^^,^^78^ While the *Plodia* major sericins show numerous Serine residues (Figures 3C and 3D), and the sericins undergo GalNAc O-glycosylation in *Bombyx*,^79^ Pgant5 is unlikely to modify sericins here as it lacks an N-terminal signal peptide (Table S8). Instead, Pgant5 may thus glycosylate intracellular factors, akin to its role in fly intestinal cells.^80^

In contrast, the MSG enzymatic factors mentioned below each carry an N-terminal signal peptide (Table S8), indicating they are all targeted to secretory organelles and play a role in the modification of silk factors. The *ERp60* gene, encoding a disulfide isomerase enzyme, is highly expressed in the MSG and may be required for the formation of covalent bonds between silk factors such as FibL and FibH.^23^ Similarly, we found the MSG-enrichment of enzymes involved in the degradation of complex lipids such as several lipase genes (*Lip1*, *Lip3*, *Lip3-like*, *Lip4*, *LipF*, *LipK*, and *LipK-like*), a glucocerebrosidase gene (*Gba1c*), and a saposin gene (*Sap-r*). Similar lipid modifiers were previously detected in the silk glands of several moths and caddisflies,^76^^,^^81^^,^^82^^,^^83^ suggesting that lipid degradation is a key aspect of silk biology in Amphiesmenoptera (i.e., Lepidoptera and Trichoptera).

### Spatial hybridization chain reaction identifies two specialized spatial domains within the Middle Silk Gland

The RNAseq dataset depicts an overview of transcriptome divergence between two exocrine tissues, secreting either the silk fiber or its coating layers. The PSG transcriptome was consistent with a specialized secretory role focused on producing the fibroin core of the silk fiber. Meanwhile, the apparent transcriptomic complexity of the MSG could reflect either the presence of multiple distinct cell types or greater molecular heterogeneity within a more homogeneous population. To assess this, we selected 11 MSG-enriched genes and profiled their spatial expression patterns using HCR.

**Figure 4:**
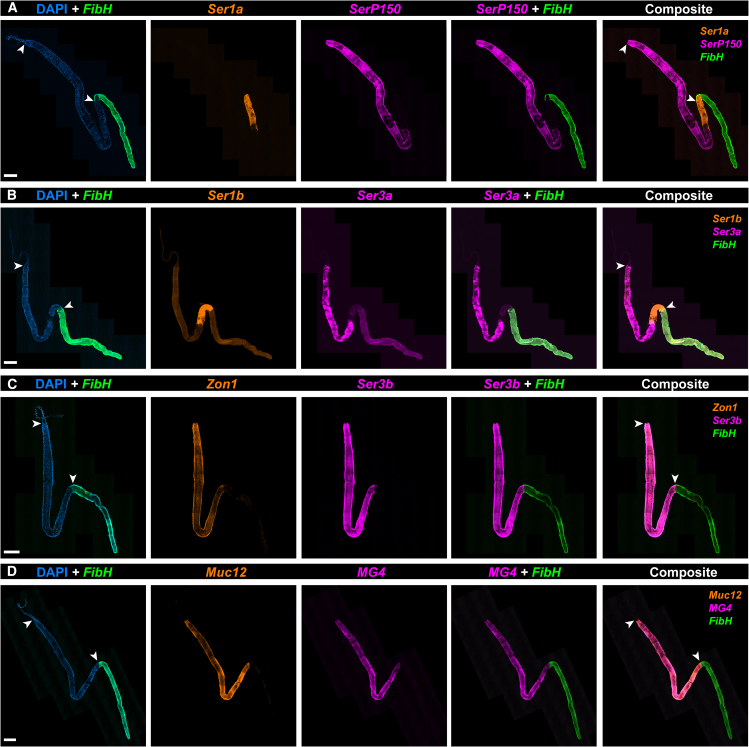
The MSG is compartmentalized into two subdomains of sericin and zonadhesin expression Whole-mount silk gland HCR stainings of 8 MSG-enriched adhesive protein factors, including 7 sericin factors (variably annotated as sericin and mucin genes) and a zonadhesin (A) *Sericin1a* (*Ser1a*), *SericinP150* (*SerP150*), and fibroin heavy chain (*FibH*) mRNA. (B) *Sericin1b* (*Ser1b*), *Sericin3a* (*Ser3a*), and *FibH* mRNA (C) *Zonadhesin1* (Zon1), *Sericin3a* (*Ser3a*), and *FibH* mRNA (D) *Mucin12* (*Muc12*), *MG4*, and *FibH* mRNA. Arrowheads: ASG/MSG (A-D: top) and MSG/PSG boundaries (A-D: bottom). Scale bars: A-D = 500 μm.

The expression of the tandem-duplicate genes, *Ser1a* and *Ser1b*, is restricted to the posterior region of the MSG directly adjacent to the anterior PSG as defined by *FibH* expression (Figures 4A and 4B). HCR also found expressions of *SerP150* and *Ser3a* are limited to the anterior region of the MSG (Figures 4A and 4B). In contrast, *Mucin12*, *MG4*, *Ser3b* (a *Ser3a* paralog), and *Zonadhesin1* were expressed throughout the entire MSG compartment (Figures 4C and 4D). These results suggest that the MSG consists of two cell types, each restricted to two spatial domains: MSG-P, a small posterior compartment specialized in the expression of inner coating layer proteins (e.g., Ser1a/Ser1b); and MSG-A, a longer, more anterior compartment of the MSG that secretes major sericins (*Ser3a*/*Ser3b*, *MG4*, *SerP150*, and *Mucin12*) and *Zon1*.

### Expression of antimicrobial and conditioning factors

**Figure 5:**
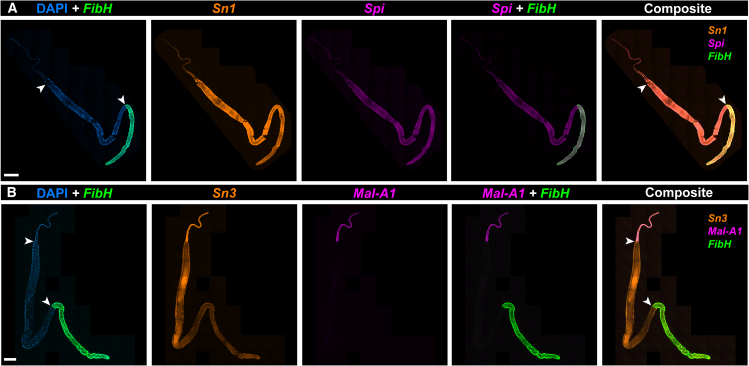
HCR detection of *seroins* and *Mal-A1* expression (A) Whole-mount silk gland HCR staining of *Seroin1* (*Sn1*), *Silk protease inhibitor* (*Spi*), and *FibH* mRNA. (B) HCR staining of *MaltaseA1* (*Mal-A1*), *Seroin3* (*Sn3*), and *FibH* is shown in a whole silk gland. Arrowheads: ASG/MSG (A-B: top) and MSG/PSG boundaries (A-B: bottom). Scale bars: A-B = 500 μm.

We broadly define silk conditioning factors as the molecules that are secreted by the silk gland to modify the properties of silk without playing a structural role. Of particular interest, seroins and serine protease inhibitors (serpins) act as broad-spectrum antibacterial and antifungal agents involved in preventing silk degradation.^10^^,^^35^^,^^84^ In addition to the high expression of the seroin *Sn1*, RNAseq also detected the seroin gene *Seroin3* (*Sn3*) and the serpin *Silk protease inhibitor* (*Spi*) genes (Figures 3B and 3C), previously studied in *Galleria* and *Ephestia*.^10^^,^^76^ HCR assays show that *Seroin1* (*Sn1*) is expressed throughout the PSG and MSG, and *Sn3* is high in the ASG and low in the MSG (Figure 5). While the function of these factors in microbial inhibition remains to be tested, gene expression patterns support the finding that *Plodia* silk contains bioactive antimicrobial properties.^85^

Lastly, RNAseq detected the expression of *Maltase-A1* (*Mal-A1*), a gene encoding an enzyme likely involved in the hydrolysis of maltose into glucose (Figures 3A and 3B). As this putative function is intriguing, we sought to profile its expression with HCR and found it to be specifically expressed in the ASG (Figures 5B and 5C). This glucosidase enzyme includes an N-terminal signal peptide, suggesting it is actively secreted in the ASG lumen. We speculate that this enzyme may participate in glycoprotein maturation by modifying the carbohydrates associated with silk proteins. Alternatively, it could be secreted into the outer silk coating, acting as a pre-digestive enzyme, facilitating larval feeding in starch-rich environments.

## Discussion

### A roadmap for gene expression analyses of insect exocrine tissues

**Figure 6:**
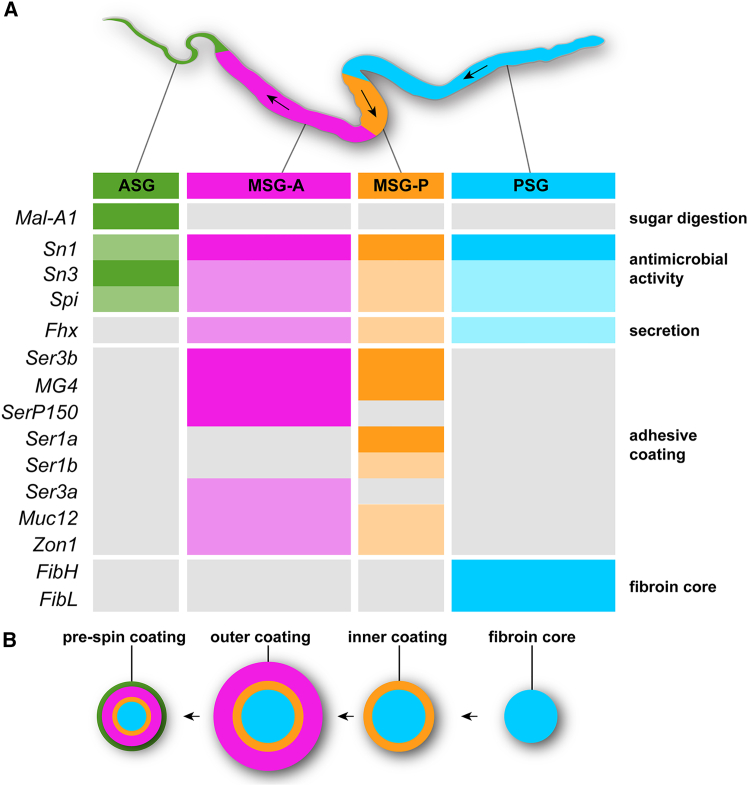
Regionalized expression of major *Plodia* silk factors defines four specialized exocrine tissues (A) Summary of the HCR expression data for 11 silk factors. Color rectangles highlight regions of expression (full color: strong HCR signal, or TPM >10,000 in MSG/PSG; dimmed colors: faint HCR signal or TPM <10,000); gray rectangles indicate low expression (no HCR signal, or TPM <2.5 in MSG/PSG). Arrows: direction of silk processing. (B) Schematic view of the silk fiber, with successive layers of coating (from right to left) secreted along the specialized silk gland regions.

As crucibles of biochemical innovation, insect exocrine tissues may offer an untapped reservoir of bioactive compounds of translational importance in biotechnology, medicine, or agriculture. In this work, we used bulk RNAseq to profile gene expression in silk glands that were split at their approximated PSG/MSG boundary and then refined the expression patterns of putative silk factors with a cellular spatial resolution, thereby identifying the major bioadhesive proteins secreted in the *Plodia* silk outer layer, as well as seroins that may confer its antimicrobial properties.^35^^,^^36^^,^^85^ In combination with the availability of reliable transcriptome or genome annotations in a given species, this strategy offers a powerful way to decipher the molecular biology of exocrine glands across insects. Indeed, secreted genes can be identified by the presence of N-terminal signal peptides, and our data suggest that peptidic components of biological importance (e.g., fibroins and sericins) are expressed at high levels in secretory cells (Figure 6). In Lepidoptera alone, more than 34 exocrine tissues have been described—most of them awaiting molecular characterization— including venom glands, myrmecophilic organs, and salivary glands that provide specialized interactions with predators, symbionts, and host plants.^86^ In addition, other holometabolous insects such as wasps, sawflies, and glowworms also use labial glands to produce diverse silks that differ in composition from the lepidopteran silk^1^; gene expression profiling combining RNAseq and HCR could depict the modalities of convergence in the molecular toolkits underlying silk production across independently evolved insect lineages.

### Secretory specializations and cell-type regionalization of the silk gland

We generated deep transcriptomes of *Plodia* last instar MSG and PSG and uncovered the identity and spatial domains of major silk factors, including fibroins, sericins, and seroins (Figure 6A), some of which are sequentially secreted to constitute the concentric layers of the silk fiber and its coating (Figure 6B). These data suggest that the *Plodia* silk gland mainly consists of four regions and exocrine cell types: PSG cells specialized in core fibroin secretion, two successive types of MSG cells each secreting a distinct mixture of adhesive proteins, and ASG cells.

In addition, we started to uncover the regionalized expression of antimicrobial factors such as seroins and serine-protease inhibitors, suggesting that the deposition of silk conditioning factors can be assigned to distinct layers of the fiber. In conjunction with previous expression studies in other lepidopteran silk glands,^10^^,^^35^^,^^37^^,^^76^^,^^87^^,^^88^ these data suggest that the expression of bioactive molecules involved in the prevention of silk degradation is an important conserved quality of lepidopteran silk. Future studies of this process could extend to other seroins and serpins detected in the dataset and assess the effects of larval stage and microbial exposure on expression patterns.

Unexpectedly, HCR spatial assays identified *Mal-A1* as a specific marker of the ASG. This result highlights a limitation of our RNA-seq study: because silk glands were sectioned at the MSG/PSG boundary, these samples also included the comparatively smaller ASG gland, and some of the MSG-enriched transcripts described in this study may turn out to be enriched in the ASG instead. Due to the relatively small size of the ASG tissue compared to the rest of the MSG (Figures 1 and S1), we can infer that ASG-specific transcripts are largely obscured by the MSG contribution to this dataset, and further studies of the elusive functions of the ASG would benefit from RNAseq resampling combined with HCR spatial assays.

### Spatial homology between the silk gland sub-domains of *Plodia andBombyx*

The overall morphology and exocrine function of silk glands appears conserved across many silk-spinning lepidopterans, including in Bombycoidea,^5^^,^^28^^,^^40^^,^^83^^,^^89^^,^^90^ Psychidae,^41^ Nolidae,^91^ and Plutellidae,^92^^,^^93^ with a PSG portion dedicated to the secretion of fibroins, and an MSG portion involved in the coating of outer layers of adhesive proteins. RNAseq and HCR spatial analyses of silk factor expression confirm this big picture in *Plodia* (Figure 6), complementing previous analyses in Pyralidae.^10^^,^^12^^,^^39^^,^^94^

We found that the tandem duplicate genes *Ser1a* and *Ser1b* are specifically expressed in a short domain, previously known as the rear-MSG in Pyralidae.^10^ Using qPCR in silk gland sections of the pyralid *Ephestia*, Wu et al. previously found a similar pattern with both *Ser1a* and *Ser1b* showing rear-MSG enrichment and proposed that *Sericin1* genes, characterized by a signature CxCx motif in their C-terminal region, highlight the conservation of this compartment with *Bombyx* and beyond.^29^ Microsynteny analyses further support the homology of *Plodia Ser1a* and *Ser1b* with *Bombyx Ser1*, and the HCR mRNA expression of *Plodia Ser1a* and *Ser1b* was also reminiscent of the spatial expression of *Ser1* in *Bombyx* fourth instar larvae, in a subsection of the MSG immediately anterior to the PSG that later extends to the whole MSG during the fifth instar^29^^,^^30^ (Figure S3) In *Plodia*, restricted expressions of *SerP150* and *Ser3a* define a long, anterior domain of the MSG (here dubbed MSG-A), while both MSG-A and MSG-P expressed *Ser3b*, *MG4*, *Muc12*, and *Zon1*. We did not find molecular evidence that this MSG-A region can be subdivided into two portions, as proposed for *Galleria* wax moths^76^ As this is the first time that these adhesion genes are characterized in whole-mount expression assays, HCR experiments are needed to assess the sub-regionalization of the MSG in other species.

In summary, we can affirm that *Bombyx* and *Plodia* silk glands share a homologous PSG, a *Ser1*-positive MSG-P compartment involved in the secretion of the silk inner coating layer,^10^^,^^29^^,^^30^ and an MSG-A region involved in the secretion of the outer coating layer, marked by spatially restricted domains of *SerP150* and *Ser3a* in *Plodia*. Of note, sericin expression and alternative splicing profiles can vary across stages in the silkworm, as cocoon-specific silk differs from the silk spun during earlier larval stages.^95^^,^^96^ Our findings in *Plodia* set the stage for broader surveys that may reveal both the conserved ground plan and lineage-specific innovations underlying silk gland evolution.

### Limitations of the study

In the RNAseq experiment, we used a single, approximate section between the MSG and PSG to separate these two tissues. Thus, the underlying MSG and PSG transcriptomes can include small contributions from the other tissue to gene expression. MSG transcriptomes also include the ASG.

## Resource availability

### Lead contact

Requests for further information, resources, and reagents should be directed to the lead contact, Arnaud Martin (arnaud@gwu.edu).

### Materials availability

Detailed procedures for immunofluorescence, fluorescent dye, and HCR stainings can be found in the Open Science Framework repository.^97^

### Data and code availability

• The raw reads from RNA sequencing have been deposited in the NCBI Sequence Read Archive under BioProject PRJNA1241317. The manually curated *ilPloInte3.2* NCBI RefSeq annotation GTF file and gene list can be found in Data S1 and Table S9, respectively.
• All code associated with this project can be found in the GitHub repository: https://github.com/jasalq/Plodia_Silk_RNAseq.
• Detailed tables of the results from the differential expression analyses can be found in Tables S2, S3, S4, S5, and S6.

## Acknowledgments

We thank Donya Shodja and Christa Heryanto for their support rearing *Plodia*; Luca Livraghi for guidance on HCR protocol optimization; Patricia Hernandez and Aleksandar Jeremic for providing access to confocal microscopes; Anastas Popratiloff and personnel of the GW Nanofabrication and Imaging Center (GWNIC) for support with spinning disk confocal microscopy. Micro-computed tomography was conducted at the Research Service Centers of the Herbert Wertheim College of Engineering at the University of Florida with the valuable assistance of Gary Scheiffele. This work was funded through a collaborative IntBIO grant from the 10.13039/100000001National Science Foundation (awards MCB-2217156 to AM and MCB-2217159 to WLS) and a 10.13039/100000002National Institutes of Health 10.13039/100000057National Institute of General Medical Sciences Maximizing Investigators’ Research Award to WLS (NIH 10.13039/100000057NIGMS R35-GM147041). Microscopy equipment at the GWNIC was funded by the award NIH 1S10OD010710-01.

## Author contributions

Conceptualization and writing – original draft: J.D.A. and A.M.; investigation: J.D.A., M.B., L.E.E., W.L.S., and A.M.; formal analysis: J.D.A.; methodology: J.D.A and M.B.; visualization: J.D.A., M.B., L.E.E., and A.M.; funding acquisition and supervision: W.L.S. and A.M.; and writing – review and editing: L.E.E and W.L.S.

## Declaration of interests

The authors declare no conflict of interest.

## STAR★Methods

### Key resources table

**Table:** 

REAGENT or RESOURCE	SOURCE	IDENTIFIER
**Antibodies**		

Alexa Fluor® 647 AffiniPure™ Donkey Anti-Mouse IgG (H + L)	ThermoFisher Scientific	AB_2340863
Alexa Fluor® 488 AffiniPure™ Donkey Anti-Rabbit IgG (H + L)	ThermoFisher Scientific	AB_2313584
Beta-Catenin rabbit polyclonal antibody	Sigma Aldrich	Cat#C2206-25UL
Fibrillarin mouse monoclonal antibody	Cytoskeleton Inc.	Cat#AFB01

**Chemicals, peptides, and recombinant proteins**		

Bovine Serum Albumin (BSA)	ThermoFisher Scientific	Cat#J65788.09
4′,6-diamidino-2-phenylindole, dihydrochloride (DAPI)	ThermoFisher Scientific	Cat#D1306
EDTA, 0.5 M, pH 8.0	Bioland Scientific	Cat#EDTA01
EGTA, 0.5 M, pH 8.0	bioWorld	Cat#40520008-1
Formaldehyde, 37%	Sigma Aldrich	Cat#252549-500 ML
Formalin, buffered, 10%	Fisher Scientific	Cat#SF100-4
Glycerol, ≥99.0%	Sigma Aldrich	Cat#G5516
Low EDTA TE (1x) buffer, pH 8.0	Quality Biological	Cat#351-324-721
Lugol’s iodine solution	Sigma Aldrich	Cat#L6146-1L
Methanol, 100%	Lab Alley	Cat#MAL-1GAL
Methanol-free Paraformaldehyde, Aqueous Solution EM Grade, 16%	Electron Microscopy Sciences	Cat#15170
PBS, 10x	UFC Bio	Cat#BPBS74-10X
Phalloidin-Oregon Green 488, 300 U	ThermoFisher Scientific	Cat#O7466
SlowFade™ Gold Antifade Mountant	ThermoFisher Scientific	Cat#S36937
SSC, 20x	Quality Biological	Cat#351-003-101
Triton X-100	Sigma Aldrich	Cat#9036-19-5
Tween 20	Bioworld	Cat#42030016-1
Wheat Germ Agglutinin (WGA)-Alexa Fluor 555, 1 mg/mL	ThermoFisher Scientific	Cat#W32464

**Critical commercial assays**		

HCR v.3.0 Alexa Fluor 488 Amplifier (B1)	Molecular Instruments	N/A
HCR v.3.0 Alexa Fluor 647 Amplifier (B3)	Molecular Instruments	N/A
HCR v.3.0 Alexa Fluor 594 Amplifier (B5)	Molecular Instruments	N/A
HCR v3.0 Hybridization Buffer, Probe Wash Buffer, Amplification Buffer	Molecular Instruments	N/A

**Deposited data**		

ilPloInte3.2 Plodia interpunctella reference genome assembly and annotation	NCBI Refseq	NCBI: GCF_027563975.2
*Plodia interpunctella* manually curated genome annotation	This study	Data S1
*Plodia interpunctella,* RNAseq transcriptomes of fifth instar larval silk glands (ASG/MSG, PSG), salivary glands, and heads	This study	NCBI: PRJNA1241317

**Experimental models: organisms/strains**		

*Plodia interpunctella,* bFog laboratory strain	This study	NCBI:txid58824

**Oligonucleotides**		

Oligonucleotides for HCR, see Table S1	This study	N/A

**Software and algorithms**		

Adobe Illustrator and Adobe Photoshop	Adobe	URL: https://www.adobe.com/
Blast+ v.2.16.0+	Camacho et al. 2009	URL: https://blast.ncbi.nlm.nih.gov/doc/blast-help/downloadblastdata.html
Fastp v.0.21.0	Chen et al. 2018	URL: https://github.com/OpenGene/fastp
FastQC v.0.11.8	GitHub/S-andrews	URL: https://github.com/s-andrews/FastQC
FIJI	Schindelin et al. 2012	URL: https://imagej.net/software/fiji/downloads
Geneious Prime	Geneious	URL: https://www.geneious.com/
*Insitu* Probe Generator v.0.3.2	Kuehn et al. 2022	URL: https://github.com/rwnull/insitu_probe_generator
MAFFT	Katoh & Standley 2013	URL: https://www.geneious.com/plugins/mafft
Napari	Chiu et al. 2022	URL: https://github.com/napari/napari
Mauve	Darling et al. 2010	URL: https://www.geneious.com/plugins/mauve
OWLTools v.4.5.29 (Map2Slim)	Github/owlcollab	URL: https://zenodo.org/records/13733246
*R/clusterProfiler*	Xu et al. 2024	URL: https://bioconductor.org/packages/devel/bioc/html/clusterProfiler.html
*R/cowplot*	Wilke 2024	URL: https://CRAN.R-project.org/package=cowplot
*R/DESeq2*	Love et al. 2014	URL: https://bioconductor.org/packages/release/bioc/html/DESeq2.html
*R/dplyr*	CRAN/Wickham et al.	URL: https://cran.r-project.org/web/packages/dplyr/index.html
*R/ggdendro*	CRAN/de Vries & Ripley	URL: https://cran.r-project.org/web/packages/ggdendro/index.html
*R/ggplot2*	Tidyverse/Hadley Wickham	URL: https://ggplot2.tidyverse.org
*R/gridExtra*	CRAN/Baptiste Auguie	URL: https://cran.r-project.org/web/packages/gridExtra
*R/ontologyIndex*	CRAN/Daniel Greene	https://cran.r-project.org/web/packages/ontologyIndex/index.html
*R/pals*	CRAN/Kevin Wright	URL: https://cran.r-project.org/web/packages/pals/index.html
*R/patchwork*	CRAN/Thomas Lin Pedersen	URL: https://cran.r-project.org/web/packages/patchwork/index.html
*R/readxl*	CRAN/Wickham & Bryan	URL: https://cran.r-project.org/web/packages/readxl/index.html
*R/reshape2*	CRAN/Hadley Wickham	URL: https://cran.r-project.org/web/packages/reshape2/index.html
*R/stringr*	CRAN/Hadley Wickham	URL: https://cran.r-project.org/web/packages/stringr/index.html
*R/tidyverse*	Wickham 2016	URL: https://www.tidyverse.org/
Samtools v.19	Li et al. 2009	URL: https://github.com/samtools/samtools
SignalP v6.0	Teufel et al. 2022	URL: https://services.healthtech.dtu.dk/services/SignalP-6.0/
STAR v.2.7.11b	Dobin et al. 2016	URL: https://github.com/alexdobin/STAR
Subread v.2.0.8 (featureCounts)	Liao et al. 2014	URL: http://subread.sourceforge.net
W-IQ-TREE	Trifinopoulos et al. 2016	URL: http://iqtree.cibiv.univie.ac.at/

**Other**		

Black Enameled Pins, Size 1	Pin-It Entomological Supply	N/A
Copper Wire Mesh,100 × 100 Mesh, 0.0045″ Diameter Wire	Small Parts	Cat#CU-100-0045-01
Detailed methods for this paper	This study	OSF: https://doi.org/10.17605/OSF.IO/3PZ6C
Dumont #4 Forceps	Fine Science Tools	Cat#11242-40
LocknLock Rectangular, 350 mL	LocknLock	Cat#HPL806
Mason Jar, 16 oz	Ball	Cat#52150000
RNase Away, 475 mL Spray Bottle	Fisher Scientific; Research Products International Corp	Cat#50-197-8158
Small Dissection Petri Dish, Clear, 50 mm Dia x 17 mm H	Fisher Scientific; Living Systems Inc	Cat#NC1257845
Stainless Steel Cup Holder	Da Vinci	Cat#B06W2JBLJJ
Steel Woven Wire Cloth Disc, 40 × 40 Mesh, 2–9/16″ Diameter	McMaster-Carr	Cat#2812T43
Super HT PAP Pen, Mini, 2.5 mm Tapered Tip	RPI	Cat#195506
Vannas Spring Scissors - 2.5 mm	Fine Science Tools	Cat#15000-08

### Experimental model and study participant details

The wild-type *bFog* laboratory strain of *P. interpunctella* was reared in the laboratory from egg to adulthood in a growth chamber at 28°C with 60–80% relative humidity and a 14:10 h light:dark cycle.^54^ Rearing methods are available online on the Open Science Framework (OSF) repository.^97^ Briefly, egg laying was induced by CO_2_ narcosis of adult stock in an oviposition jar, and a weight boat containing 10–12 mg eggs was placed in a rearing container containing 45–50 g of wheat bran diet with 30% glycerol. At 28°C, this life cycle spans 29 days from fertilization to a reproductively mature adult stock.

### Method details

#### Micro-computed tomography

A fifth instar larva was fixed in 10% phosphate buffered formalin overnight before the skin layer was nicked by needle pins and then returned to fixative for 48 h. Fixed larvae were submerged in Lugol’s iodine contrast solution for 1 week and rinsed in ultrapure water to remove excess contrast solution directly prior to scanning. The stained specimen was scanned on a GE Phoenix v|tome|x m 240 CT scanner (GE HealthCare Technologies, USA) equipped with a 180 kV transmission tube and diamond target with a 4.98 μm voxel size, 120 μA current, and 80 kV voltage. 3D volume files were analyzed and rendered with Volume Graphics VGStudio Max v2024.2 software suite (Volume Graphics, GER). Silk gland segmentation, refinement, and smoothing was completed with VGStudio Max’s segmentation tools on reconstructed 3D volumes.

#### Antibody and fluorescent dye stainings

Protocols for the dissection, immunofluorescent staining, and dye stainings of *Plodia* silk glands are described online on the OSF repository.^97^ Briefly, whole silk glands were dissected from wandering fifth instar larvae. For both immunofluorescent and dye stainings the samples were fixed in a 4% methanol free formaldehyde solution for 20 min and then washed three times for 5 min in PBT at room temperature. For immunofluorescent staining, the samples were washed for four 5 min washes in PT and then blocked in a PT-BSA solution at room temperature. Samples were incubated in the primary antibodies solution overnight at 4°C. After incubation two quick washes and three 8 min washes in PT were performed. Samples were then blocked again in PT-BSA for 10 min at room temperature before the secondary antibody solution was added for incubation at room temperature for 2 h. Final washes in PT (two quick and four 15 min washes) were performed before a solution of 50% glycerol with 1.0 μg/mL DAPI (pH 7.4) was added to incubate either for 30–60 min at room temperature or overnight at 4°C. Samples were mounted in 70% Glycerol (diluted in PBS with pH 7.4) and imaged.

For dye stainings, after the samples were originally washed in PBT, a diluted solution of WGA-Alexa 555 in 1X PBS was added to the samples and left to incubate for 1 to 2 h in the dark at room temperature. Three 5 min washes in PBT and two quick washes with PT were then performed before staining in a diluted solution of Phalloidin (Oregon Green)-Alexa 488 in PT for either 2 to 4 h at room temperature or 18 to 24 h at 4°C. After staining, three quick washes and three 10 min washes with PT were performed at room temperature and a solution of 50% glycerol with 1.0 μg/mL DAPI (pH 7.4) was added to incubate either for 30–60 min at room temperature or overnight at 4°C. Samples were mounted in 70% Glycerol diluted in PBS under a #1.5 glass coverslip before imaging.

#### Fluorescent in situ hybridization chain reaction (HCR)

A modified version of the third-generation *in situ* hybridization chain reaction protocol was performed,^66^^,^^98^ and is fully described with solution recipes on the OSF repository.^97^ Briefly, probes were designed for target genes using the *insitu_probe_generator* software.^99^ Silk glands were dissected in cold 1X PBS and fixed in a 3.7% formaldehyde solution for 20 min at room temperature. After four 5 min washes in PBT the samples were dehydrated progressively in cold methanol diluted in PBS until a final concentration of 100% methanol was reached. Some samples were stored in 100% methanol at −20°C before continuing the protocol. All samples, freshly dehydrated or stored, were rehydrated progressively by adding decreasing concentrations of cold methanol diluted in PBS. After three 5 min washes in PBT, samples were placed in 200 μL of pre-warmed probe hybridization buffer and placed at 37°C for 30 min while shaking. Samples were then incubated while shaking overnight at 37°C while in probe hybridization solution. After the incubation, four 15 min washes in probe wash buffer were performed at 37°C. At room temperature, three 5 min washes in 5 x SSCT were performed while shaking and samples were pre-amplified in amplification buffer for 30 min-1 hr. During incubation, the amplifier solution was prepared by heating 4 μL of each amplifier for 90 s at 95°C and allowing them to cool for 30 min at room temperature in the dark. The amplifiers were then added to 200 μL of amplification buffer and after the old amplification buffer was removed, the amplifier solution was added to the samples. Samples were incubated overnight in the dark at room temperature while shaking. Amplifier solutions were then removed from the samples and stored at −20°C for re-use. Samples were washed with 200 μL 5 x SSCT at room temperature for two 5 min washes, two 30 min washes, and one 5 min wash. After removal of solution from the last wash, samples were incubated in a solution of 50% glycerol with 1.0 μg/mL DAPI (pH 7.4) either for 30–60 min shaking at room temperature or overnight shaking at 4°C. After removal of the DAPI staining solution, samples were mounted in 70% Glycerol diluted in PBS or SlowFade Gold Antifade Mountant and imaged.

#### Confocal microscopy and image processing

Whole-gland fluorescent microscopy images were obtained with a Zeiss Cell Observer Spinning Disk confocal microscope mounted with a 10x objective (Plan-Apochromat, 0.45 NA), allowing the rapid acquisition of stitched images across the whole tissue. Fluorescent images acquired from the Spinning Disk confocal were pre-processed using the ZEN acquisition software using a shading reference approach to correct for tiling artifacts. High-magnification fluorescent microscopy views of silk glands were obtained. Stacked acquisitions were also obtained on an Olympus FV1200 confocal microscope and a Zeiss LSM 800 confocal microscope, each mounted with mounted 20x and 60x objectives. Fluorescent acquisitions were processed in FIJI and Napari.^100^^,^^101^ Adjustments of contrast limits were applied independently to each fluorescent channel. Brightfield images were acquired using a Nikon D5300 camera mounted to a Nikon SMZ800N trinocular dissecting microscope, equipped with a P-Plan Apo 1X/WF 0.105 NA 70 mm objective.

#### RNA sequencing

Tissues were obtained from fifth instar wandering larvae of the *Plodia bFog* strain.^54^ Silk glands, salivary glands, and larval heads were dissected in cold 1X PBS. Silk glands were sectioned into two segments by cutting at the boundary of the MSG-P and PSG, as defined by expression of *FibH* and *FibL* (Figures 1A, 1E, and 1F). Each tissue type was obtained from two individuals and then pooled into 2 mL tubes containing 500 μL of TRI Reagent (Zymo Research) which were stored at −80°C. This process was repeated so that there were four biological replicates for each of the four tissue types. Samples were sent for total RNA extraction, poly-A enriched library preparation, and PE150 sequencing on an Illumina NovaSeq X instrument with a target yield of 30M reads per library, outsourced to Genewiz (South Plainfield, NJ), PSG samples were sequenced twice due to a suboptimal yield in the first sequencing run, and the resulting technical replicates were combined using the *collapseReplicates* command in DESeq2. Quality of the RNA sequencing data was accessed using FastQC v.0.11.8.^102^ Adapters and PolyG tails were trimmed using *Fastp* v.0.21.0^103^ with the options *–-detect_adapter_for_pe* and *–trim_poly_g*. *FastQC* was run post-trimming to check for adapter and polyG contamination and assess post-trimming read quality. RNA sequencing reads were aligned to the *P. interpunctella ilPloInte3.2* reference genome (RefSeq: GCF_027563975.2) using *STAR* v.2.7.11b.^104^ STAR alignment was repeated with the IntronMotif output from the original run to better resolve splice junctions prior to read counting.

#### Functional annotations of *plodia* gene names

The *ilPloInte3.2* NCBI RefSeq annotation of the *P. interpunctella* genome (RefSeq: *GCF_027563975.2-RS_2024_04*) was used for RNAseq transcript mapping resource with minor modification: two consecutive transcripts (GenBank: XM_053766583.1 and XM_053766584.1) were merged to form the retained *Pi_Ser1b* gene, and we added or corrected the gene models for a total of 23 antimicrobial peptide genes using manual validations of expression evidence, signal peptides, and sequence analysis. Of note, the *ilPloInte3.2* NCBI RefSeq annotation contains automated gene and protein names that are biased toward vertebrate gene nomenclature. We took two steps to complement this functional annotation with names that would be biologically informative in analyses of insect transcriptomes. First, we curated and edited the name of 30 silk factor genes, using a nomenclature reflecting direct homology to silk factors from other pyralids species as shown in this study (Table S7; S9) and previous publications.^10^^,^^70^ Second, we also added functional annotations based on sequence similarity to *Drosophila melanogaster* genes to enrich gene names with biological knowledge from a reference insect model organism. To do this, the *ilPloInte3.2* RefSeq_protein sequences were extracted using NCBI Batch Entrez, and a BLASTp reciprocal homology search using the BLAST+ tool was performed with the translated protein sequences from the FB2025_02 Flybase release of the *D. melanogaster* genome annotation.^105^ These imputed names can be seen in the “Symbol” column of Table S9 and Data S1. Read counting was performed using *FeatureCounts* (Subread v.2.0.8)^106^ using the manually curated *ilPloInte3.2* NCBI RefSeq annotation, and gene feature list with manually edited names, respectively provided online in the GTF and TSV formats (Data S1; Table S9).

#### Sericin and seroin genes microsynteny analyses and sequence alignments

Sericin and seroin gene synteny analyses were initially conducted using the *progressiveMauve*^107^ plugin implemented in Geneious Prime (2023.0.2), using the corresponding NCBI RefSeq genomic scaffolds for *B. mori* (NC_085117), *G. mellonella* (NW_026442003), and *P. interpunctella* (NC_071322). Once the target regions were identified, homology of individual genes was tested between *P. interpunctella* and putative *B. mori* or *G. mellonella* syntenologs using reciprocal TBLASTN searches using predicted protein queries from one species, to both *RefSeq_rna* and *RefSeq_genomes* of the other species. The resulting microsynteny relationships were then manually curated in Adobe Illustrator visualizations of the annotated genomic intervals (Figures S3–S7). Homologous genes of additional species were obtained from the literature^10^^,^^24^^,^^42^^,^^70^ or using NCBI TBLASTN against Lepidoptera RefSeq transcriptomes, and aligned with MAFFT^108^ in Geneious Prime. Accession numbers for the homologous genes used can be found in Table S7. Additionally the seroin amino acid alignment matrix generated by MAFFT was used by IQ-TREE with default parameters to build a phylogenetic tree.^109^

### Quantification and statistical analysis

#### Differential gene expression analyses

The count data generated by FeatureCounts was used to perform differential expression analysis using DESeq2 in RStudio.^110^ The DESeq2 function *collapseReplicates()* was used to combine sequencing runs for the PSG samples prior to performing the analysis. The experimental design *∼ Tissue* was used to define the four tissues of interest (MSG, PSG, head, salivary gland), each with four biological replicates. The initial set of differential expressed genes (DEGs) in the MSG vs. PSG contrast is defined by an adjusted *p* < 0.05 (Table S2). In parallel, TPM (transcripts per million) values were calculated for each gene to measure relative transcript abundance within each biological replicate.^71^ First, the read counts from the unnormalized count matrix were normalized by gene length in kilobases, as calculated by FeatureCounts, to obtain an RPK (reads per kilobase) value for each gene. TPM values were then calculated per sample with the formula: TPM=RPK/scalingfactor×1e6, where the scaling factor corresponds to the sum of all RPK values within the sample. Mean TPM values for each tissue were then obtained by averaging TPM values per gene across replicates.

The *plotPCA()* function of DESeq2 was used after a variance stabilizing transformation was performed with the *vst()* function to run a principal component analysis to understand sample variation. The R package *ggplot2*^111^ was used to plot the PCA results and produce a scatterplot of gene expression divergence between tissues and relative abundance within tissues (log_2_FoldChange and log2TPM). Gene Ontology (GO) enrichment analysis was performed on the DEGs identified between MSG and PSG using *clusterProfiler*^112^ after the *P. interpunctella ilPloInte3.2* GO annotation hosted on the NCBI FTP server was mapped to the *goslim_Drosophila* GO subset using *Map2Slim*. The results of the GO enrichment analysis were visualized using *ggplot2*. The *ggplot2* package was also used to produce a heatmap plot featuring the top 229 DEGs between MSG and PSG clustered by gene expression profile similarity with a significance threshold of an adjusted *p* < 0.01 and a | log_2_FoldChange | > 3, following a variance stabilizing transformation using the function *vst()* from DESeq2. After the variance stabilizing transformation the expression data was collapsed by tissue and scaled to a *Z* score. Expression profiles by gene were clustered using hierarchical clustering by the R package *ggdendro*,^113^ and *ggplot2* was then used to visualize the final heatmap. N-terminal signal peptides among top PSG and MSG enriched genes were predicted using SignalP v6.0.^114^

## Supplemental information

Document S1. Figures S1–S7

Table S1. HCR Oligonucleotides Sequences

Table S2. DESeq2 Results Table

Table S3. Table containing the DESeq2 normalized counts for all annotated genes in the *ilPloInte3.2* genome

Table S4. Table containing data plotted in the Heatmap, related to Figure 3

Table S5. Table containing the TPM normalized counts for all annotated genes in the *ilPloInte3.2* genome

Table S6. Table containing data plotted in the scatterplot, related to Figure 3

Table S7. Accession numbers for sericin factor genes used in synteny analyses and alignments

Table S8. Amino acid and signal peptide sequences for top MSG and PSG enriched genes

Table S9. Manually curated *ilPloInte3.2* Gene List

Data S1. Manually curated *ilPloInte3.2* Genome Annotation GTF
