# Supplementary material for: Regionalization of gene expression and cell types in the silk glands of Plodia pantry moths
Source: iScience. 2025 Oct 25;28(11):113865. doi: 10.1016/j.isci.2025.113865 (PMC12661448; doi:10.1016/j.isci.2025.113865)
Supplement: Document S1. Figures S1–S7 [file mmc1.pdf]

iScience, Volume *28*

Supplemental information

Regionalization of gene expression and cell types in the silk glands of *Plodia* pantry moths

Jasmine D. Alqassar, Mathilde Biot, Lauren E. Eccles, Whitney L. Stoppel, and Arnaud Martin

Supplementary Figures and References

Figure S1: DAPI and phalloidin staining of silk gland compartments
Figure S2. RNASeq sample variation and GO enrichment analyses
Figure S3: Homology and microsynteny of *Ser1* sericin genes in Lepidoptera
Figure S4: Homology and microsynteny of *SerP150* and *Mucin12* sericin factor genes in Lepidoptera
Figure S5: Homology of *MG4* sericin factor genes in Pyraloidea
Figure S6: Homology of *Ser3* sericin genes in Pyraloidea
Figure S7: Homology and microsynteny of seroin genes in Lepidoptera

Supplementary References

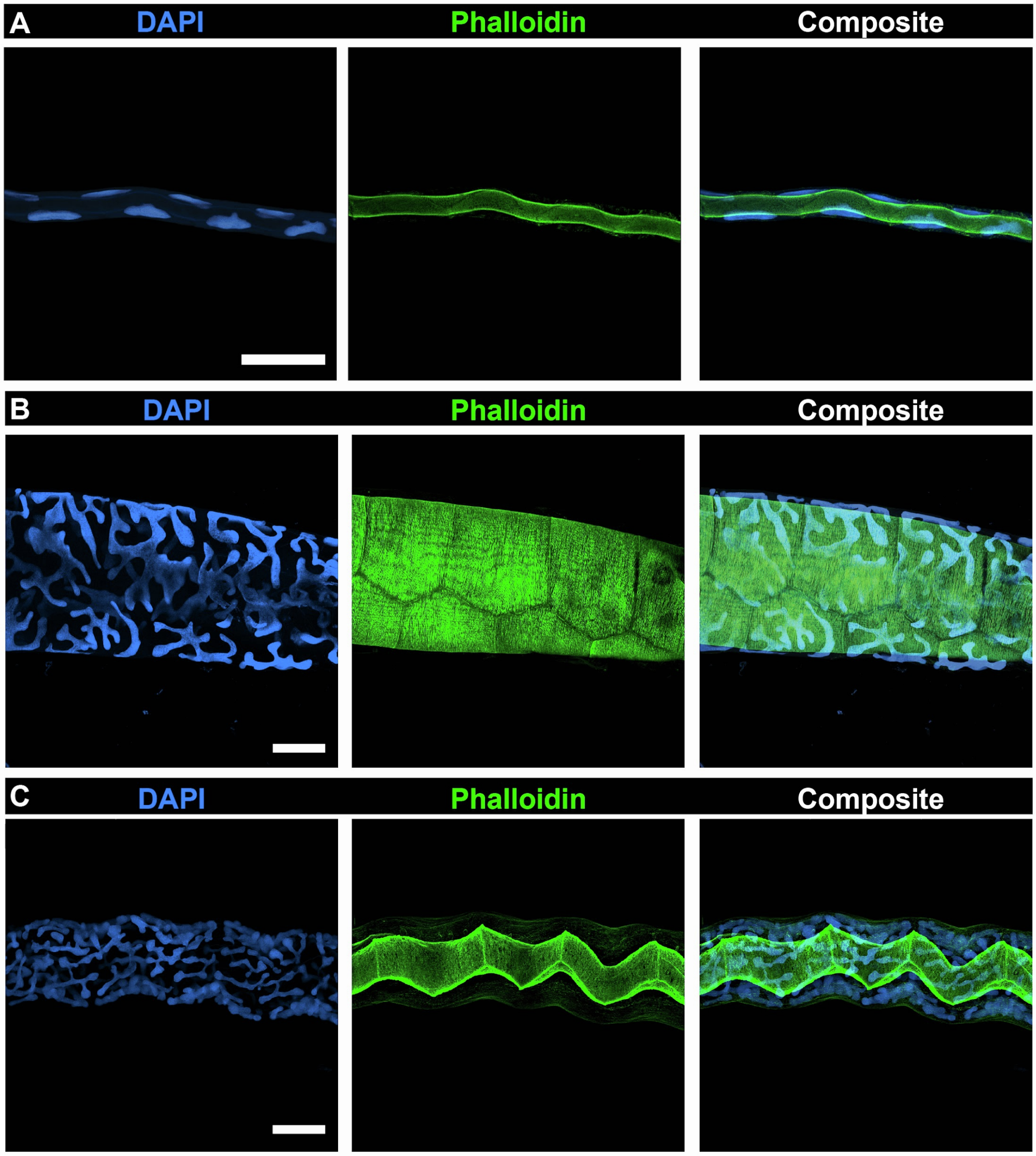

Figure S1. DAPI and Phalloidin staining reveal striking differences in cellular morphology and arrangement between compartments. A-C. DAPI and phalloidin staining of an ASG (A), MSG (B), and PSG (C). See **Video S3** for a video exploring the 3D projections of these images. The images from panel A-B are taken of the same gland while the images in panel C are taken of a different gland from an individual of the same developmental stage (wandering fifth instar). Scale bars: **A-C** = 100 μm.

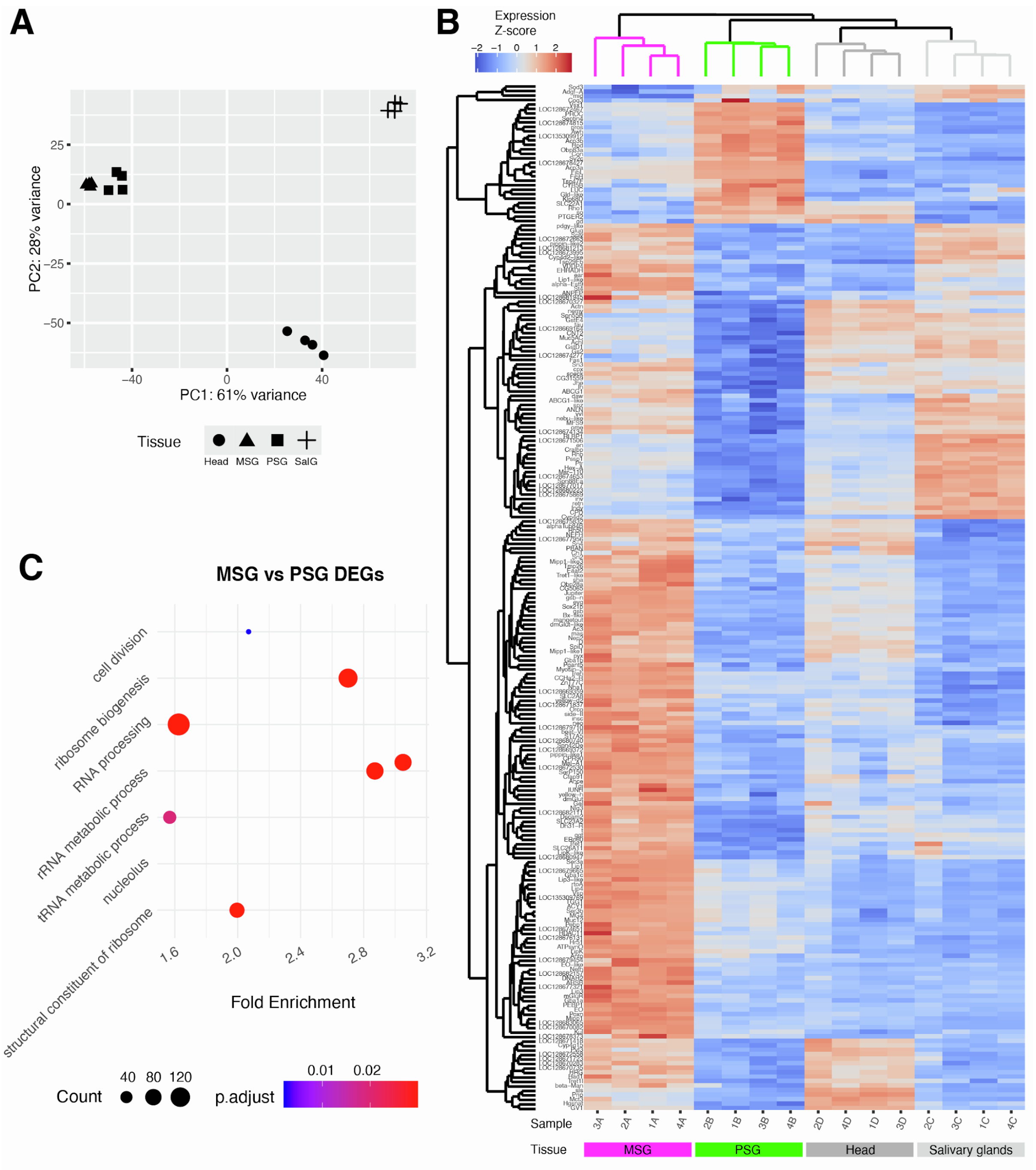

Figure S2. RNASeq sample variation and GO enrichment analyses. A. Principle component analysis showing variation between samples. . **B.** A heatmap of the top 229 DEGs between MSG and PSG (adjusted $p < 0.01$, $|\log_2\text{FoldChange}| > 3$, DESeq2 normalized counts > 300 (also seen in **Fig. 3A** with tissue average values) with expression patterns shown by sample. Dendograms delineate the hierarchical clustering of gene expression profiles (left) and samples (top). **C.** GO enrichment analysis for differentially expressed genes using the go*slim_Drosophila* reduced GO categories**.**

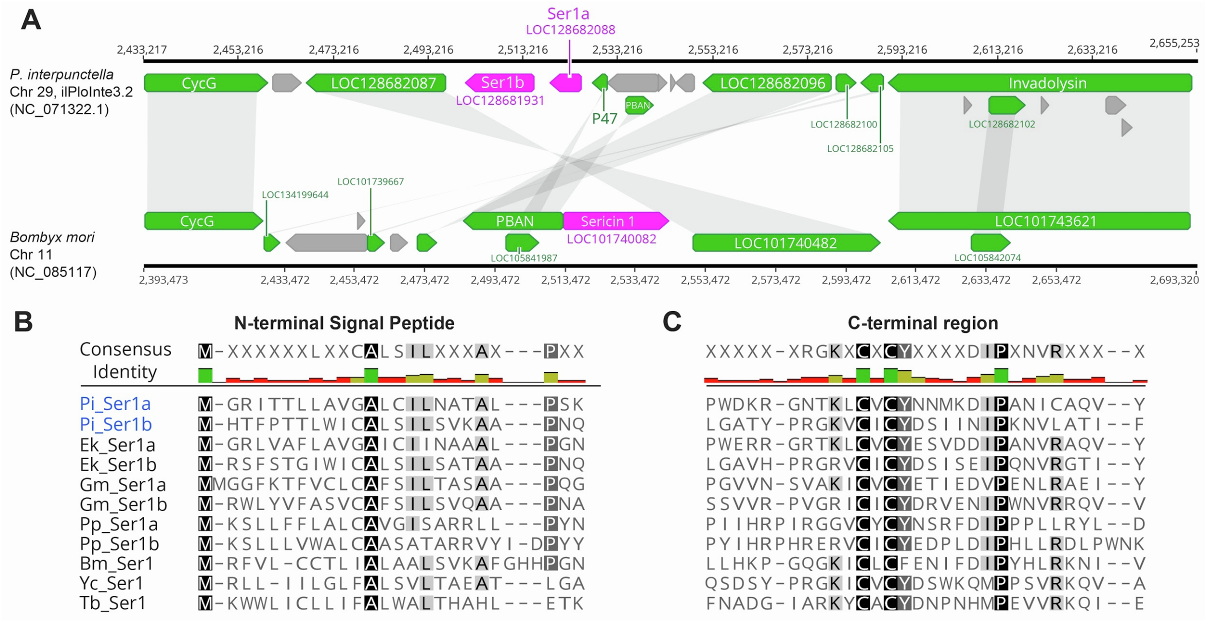

Figure S3. Homology of *Ser1* sericin gene copies across Lepidoptera. A. Synteny analysis reveals *Ser1a* and *Ser1b* genes of pyralid genomes (here on top, *P. interpunctella*) are found as tandem duplicates, within an inverted syntenic block containing the *B. mori Ser1* homolog (magenta). Grey fields indicate sequence matches using reciprocal TBLASTN between the predicted protein of a first species and the NCBI RefSeq_RNA dataset of the second species. **B-C.** Protein alignments of Ser1 syntenologs from genome annotations of lepidopteran species, largely based on previous analyses [10,41,82,91], with a focus on the N-terminal signal peptide (B) and the C-terminal region (C). The C-terminal CxCx motif is unique to this sericin orthology group. Gene and protein identifiers are listed in **Table S7**. Ek : *Ephestia kuehniella* (Pyralidae, Phycitinae), *Plodia interpunctella* (Pyralidae, Phycitinae); Gm : *Galleria mellonella* (Pyralidae, Galleriinae); Pp : *Pseudoips prasinana* (Nolidae; Chloephorinae); Bm : *Bombyx mori* (Bombycidae, Bombycinae); Yc : *Yponomeuta cagnagella* (Yponomeutidae; Yponomeutinae) ; Tb : *Tineola bisselliella* (Tineidae; Tineinae).

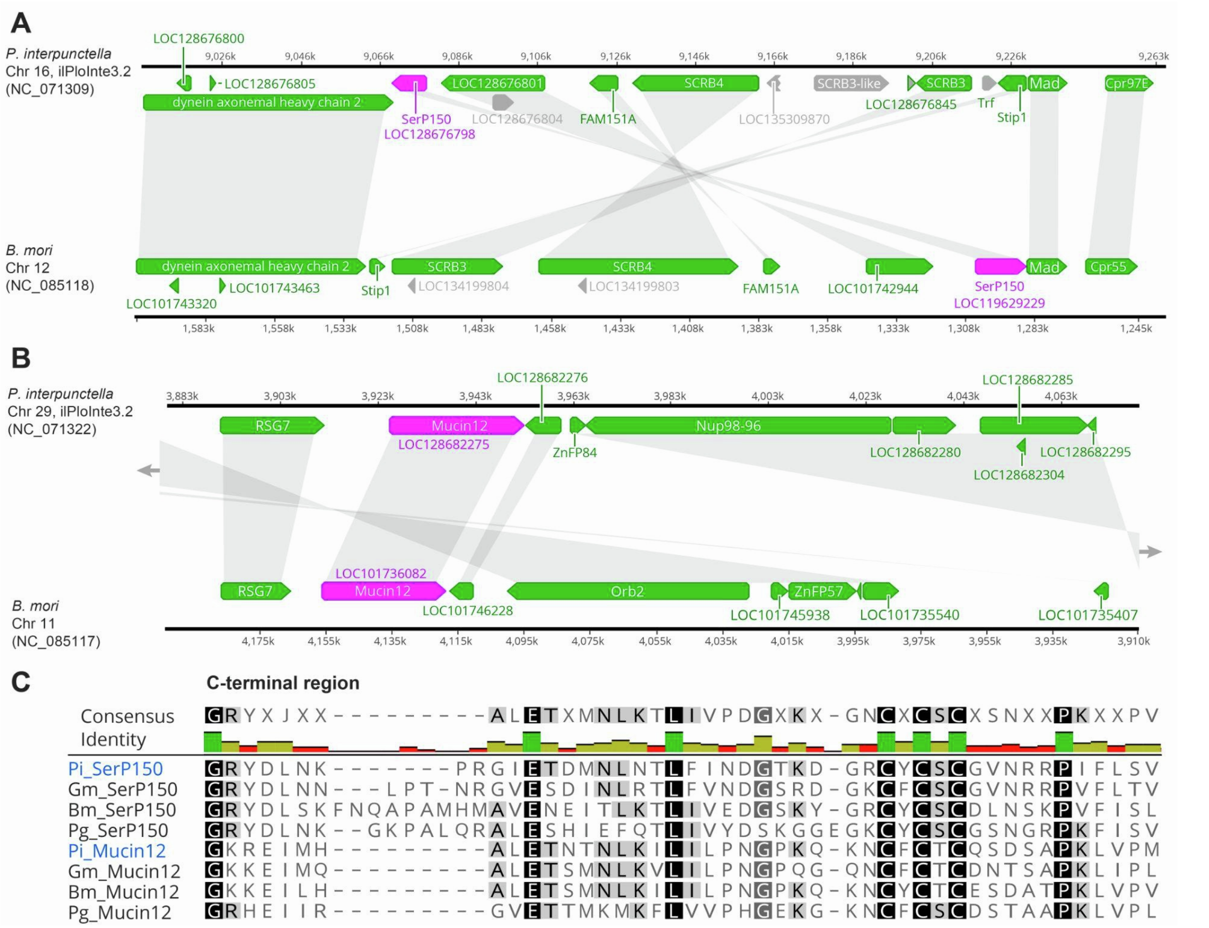

Figure S4. Homology and microsynteny of *SerP150* and *Mucin12* sericin gene orthologues in Lepidoptera. A-B. Microsyntenic relationships of the *SerP150* (A), based on a previous study[71], and *Mucin12* (B) gene regions between *P. interpunctella* (top) and *B. mori*. Grey fields indicate sequence matches using reciprocal TBLASTN between the predicted protein of a first species and the NCBI RefSeq_RNA dataset of the second species. **C.** Protein alignments of SerP150 and Mucin12 syntenologs from various lepidopteran insects, characterized by a conserved CxCxC motif in their C-terminal domains. This alignment replicates the findings of a previous study[71]. Gene and protein identifiers are listed in **Table S7**. Pi : *Plodia interpunctella* (Pyralidae); Gm : *Galleria mellonella* (Pyralidae); Bm : *Bombyx mori* (Bombycidae); Pg : *Pectinophora gossypiella* (Gelechiidae).

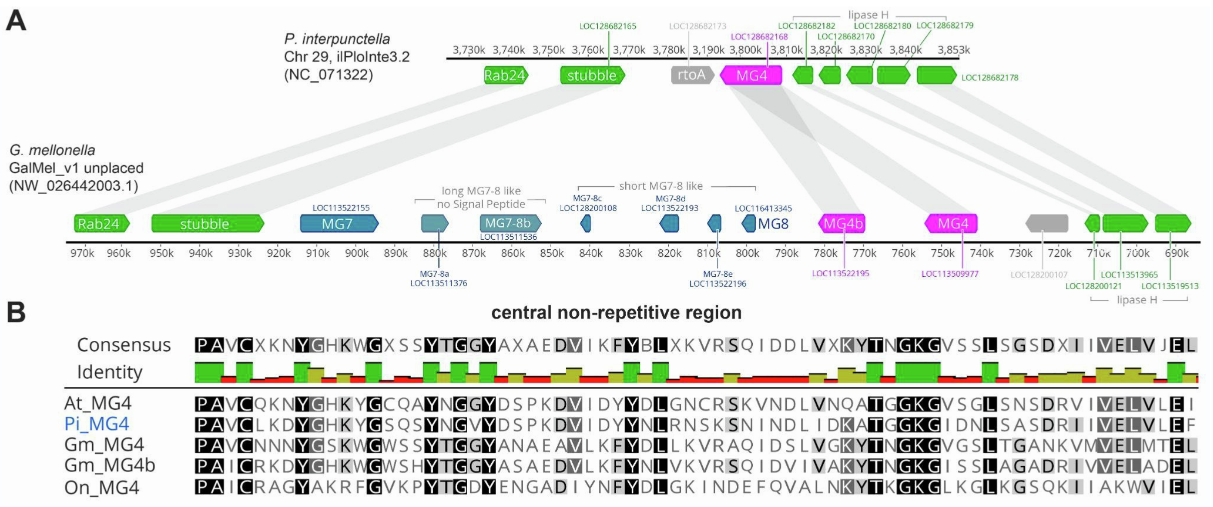

Figure S5. Homology of MG4 sericin factors in Pyraloidea. A. Synteny comparison between gene regions encompassing a single copy of the *MG4* sericin factor in *P. interpunctella* (top), and a cluster of related MSG-enriched genes specific to the *G. mellonella* genome, as previously described[10,12,115,116]. Grey fields indicate sequence matches using reciprocal TBLASTN between the predicted protein of a first species and the NCBI RefSeq_RNA dataset of the second species. *G. mellonella MG7-MG8* genes are likely paralog copies of *MG4*, consistent with an expansion of sericin genes in this species. **B.** Protein alignments of MG4 homologs from genome annotations of pyralid and crambid species (Pyraloidae), with a focus on the central domain, N-terminal to a serine-rich repeat region). Gene and protein identifiers are listed in **Table S7**. At : *Amyelois transitella* (Pyralidae, Phycitinae); Pi : *Plodia interpunctella* (Pyralidae, Phycitinae); Gm : *Galleria mellonella* (Pyralidae, Galleriinae); On : *Ostrinia nubilalis* (Crambidae, Pyraustinae).

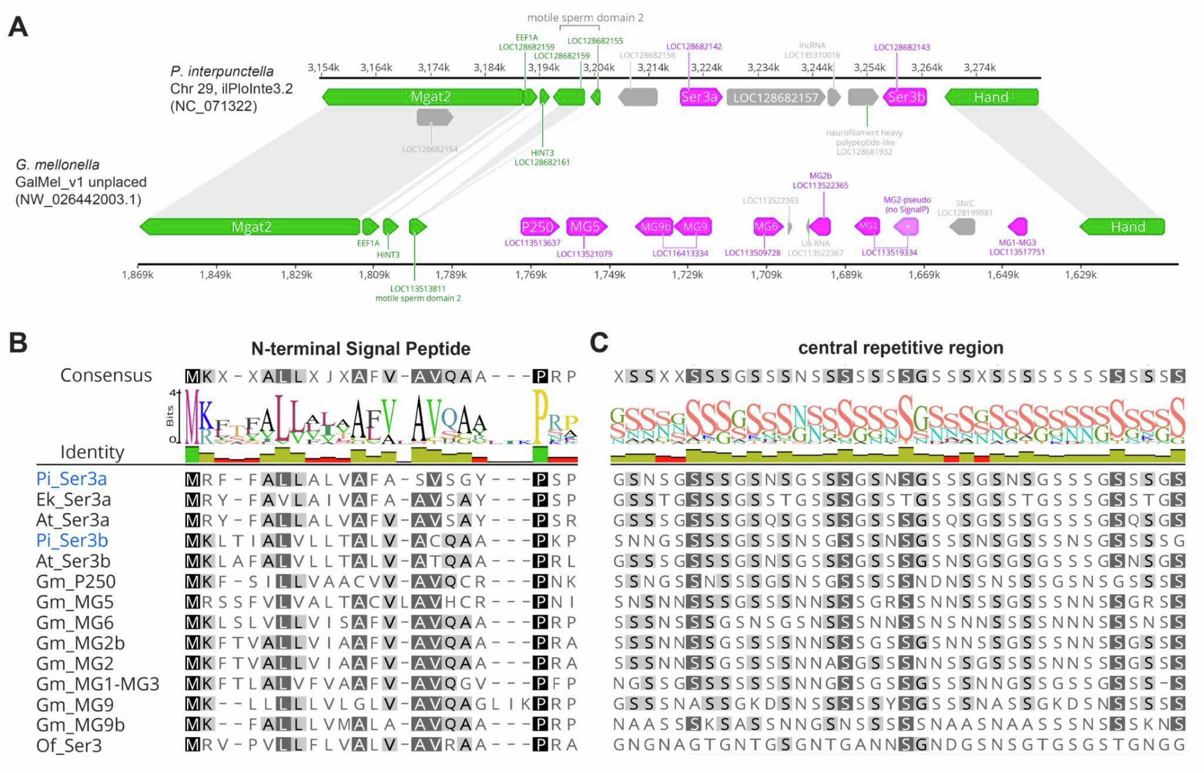

Figure S6. Homology of *Ser3* sericin factors in Pyraloidea. A. Synteny comparison between gene regions encompassing two copies of the *Sericin3* gene in *P. interpunctella* (top), hereby dubbed *Ser3a* and *Ser3b*, and a cluster of related MSG-enriched genes specific to the *G. mellonella* genome[10,12,115,116]. Grey fields indicate sequence matches using reciprocal TBLASTN between the predicted protein of a first species and the NCBI RefSeq_RNA dataset of the second species, but are only shown for flanking syntenic genes (green). Ser3 homologs (magenta) all show a high degree of similarity dominated by serine-rich repeats. **B-C.** Protein alignments of Ser3 orthologs from genome annotations of pyralid and crambid species (Pyraloidae), including copies of Ser3a previously dubbed as Ser3 in *E. kuehniella* and *A. transitella*[10], with a focus on the N-terminal signal peptide (B) and a central repetitive region (C). Gene and protein identifiers are listed in **Table S7**. At : *Amyelois transitella* (Pyralidae, Phycitinae); Ek : *Ephestia kuehniella* (Pyralidae, Phycitinae); Pi : *Plodia interpunctella* (Pyralidae, Phycitinae); Gm : *Galleria mellonella* (Pyralidae, Galleriinae); Of : *Ostrinia furnacalis* (Crambidae, Pyraustinae).

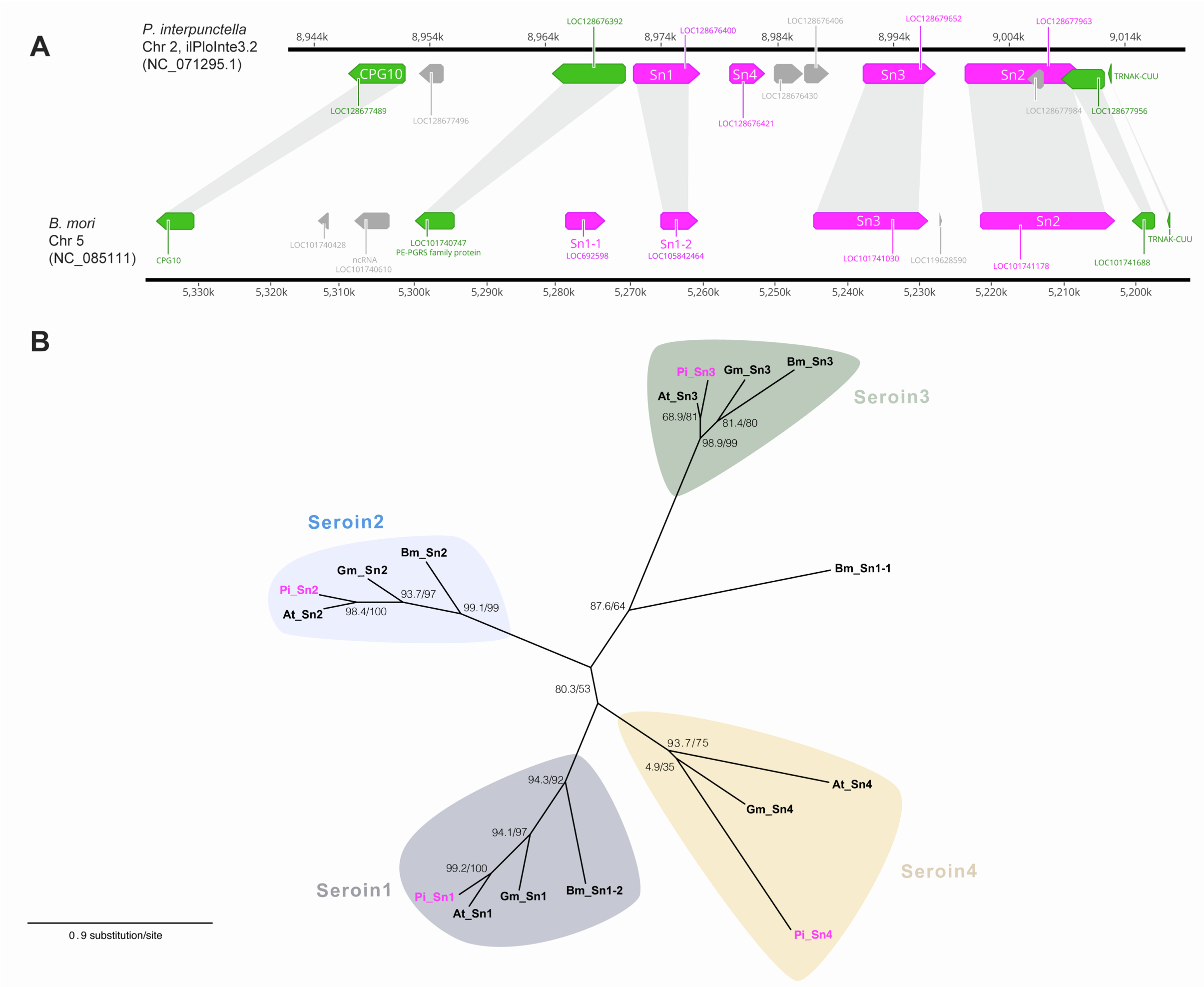

Figure S7. Homology and microsynteny of seroin genes in Lepidoptera. A. Synteny comparison between the clusters encompassing four seroin genes in *P. interpunctella* (top) and *B. mori* (bottom). Grey fields indicate sequence matches using reciprocal TBLASTN between the predicted protein of a first species and the NCBI RefSeq_RNA dataset of the second species. **B.** Maximum likelihood phylogenetic reconstruction of pyralid and *B. mori* seroin proteins, highlighting four orthology groups. Branch support is indicated by SH-aLRT % values / ultrafast bootstrap % values. Gene and protein identifiers are listed in **Table S7**. At : *Amyelois transitella* (Pyralidae, Phycitinae); Bm : *Bombyx mori* (Bombycidae, Bombycinae) ; Gm : *Galleria mellonella* (Pyralidae, Galleriinae) ; Pi : *Plodia interpunctella* (Pyralidae, Phycitinae).

Supplementary References

115. Zurovec, M., Kludkiewicz, B., Fedic, R., Sulitkova, J., Mach, V., Kucerova, L., and Sehnal, F. (2013). Functional conservation and structural diversification of silk sericins in two moth species. Biomacromolecules *14*, 1859–1866. https://doi.org/10.1021/bm400249b.

116. Zurovec, M., Yang, C., Kodrík, D., and Sehnal, F. (1998). Identification of a novel type of silk protein and regulation of its expression. J Biol Chem *273*, 15423–15428. https://doi.org/10.1074/jbc.273.25.15423.
